# Posttranslational Modifications of GLUT4 Affect Its Subcellular Localization and Translocation

**DOI:** 10.3390/ijms14059963

**Published:** 2013-05-10

**Authors:** Jessica B. A. Sadler, Nia J. Bryant, Gwyn W. Gould, Cassie R. Welburn

**Affiliations:** Institute of Molecular, Cell and Systems Biology, Davidson Building, College of Medical Veterinary and Life Sciences, University of Glasgow, Glasgow G12 8QQ, UK; E-Mails: j.sadler.1@research.gla.ac.uk (J.B.A.S.); nia.bryant@glasgow.ac.uk (N.J.B.); gwyn.gould@glasgow.ac.uk (G.W.G.)

**Keywords:** GLUT4, posttranslational modification, transport, ubiquitin, phosphorylation, SUMO

## Abstract

The facilitative glucose transporter type 4 (GLUT4) is expressed in adipose and muscle and plays a vital role in whole body glucose homeostasis. In the absence of insulin, only ~1% of cellular GLUT4 is present at the plasma membrane, with the vast majority localizing to intracellular organelles. GLUT4 is retained intracellularly by continuous trafficking through two inter-related cycles. GLUT4 passes through recycling endosomes, the *trans* Golgi network and an insulin-sensitive intracellular compartment, termed GLUT4-storage vesicles or GSVs. It is from GSVs that GLUT4 is mobilized to the cell surface in response to insulin, where it increases the rate of glucose uptake into the cell. As with many physiological responses to external stimuli, this regulated trafficking event involves multiple posttranslational modifications. This review outlines the roles of posttranslational modifications of GLUT4 on its function and insulin-regulated trafficking.

## 1. Introduction

Glucose is one of the most important energy sources for cells and is one of only three monosaccharides that are absorbed directly into the bloodstream. Optimal brain function requires glucose as an energy source, highlighting the requirement to maintain constant levels of plasma glucose [1]. When plasma glucose levels are high, glucose is removed from the bloodstream and stored as glycogen and triglycerides. When blood glucose levels fall, this glucose is released back into the bloodstream. The hormone, insulin, is critical for maintaining plasma glucose homeostasis and is released from β-cells in the islets of Langerhans in the pancreas in response to elevated plasma glucose levels. Insulin increases glucose transport into muscle and into adipose tissue. This, in turn, clears excess glucose from the bloodstream, maintaining plasma glucose levels within the normal range.

**Figure 1:**
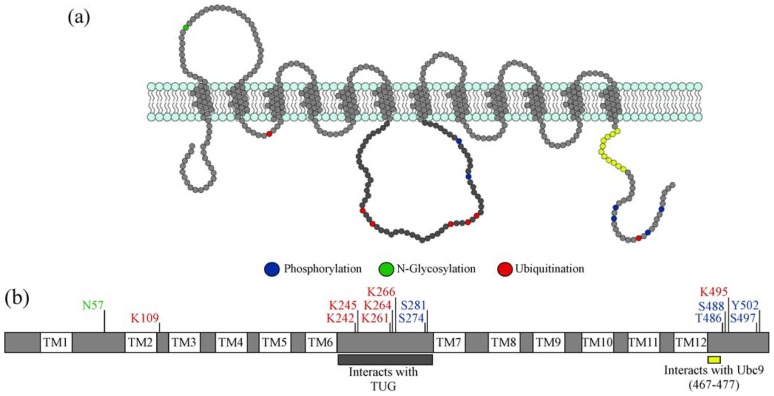
(**a**) Schematic of GLUT4, its domains and post-translational modification sites; (**b**) Map of known and putative post-translational modification sites of GLUT4 and interaction domains related to post-translational modifications. Amino acids labelled in blue may undergo phosphorylation. Phosphorylation has only been confirmed for serine 274 and serine 488. The amino acid labelled in green undergoes *N*-Glycosylation. The amino acids labelled in red are interchangeable ubiquitination sites. The dark grey bar indicates the region thought to interact with the Tether containing UBX domain (TUG); the specific amino acids involved are currently unknown. The bar in yellow indicates the amino acids shown to interact with Ubc9 (residues 467–477). The exact site of GLUT4 palmitoylation and SUMOylation are currently unknown.

The predominant insulin sensitive glucose transporter in fat cells is glucose transporter type 4 (GLUT4) [2,3]. All facilitative glucose transporter isoforms, of which 13 have been identified to date, share a similar protein structure. All possess 12 transmembrane domains, a large cytosolic loop between helices VI and VII and an exofacial loop between helices I and II; see Figure 1 [4]. The transmembrane domains have been identified as being important for glucose transport [5].

**Figure 2:**
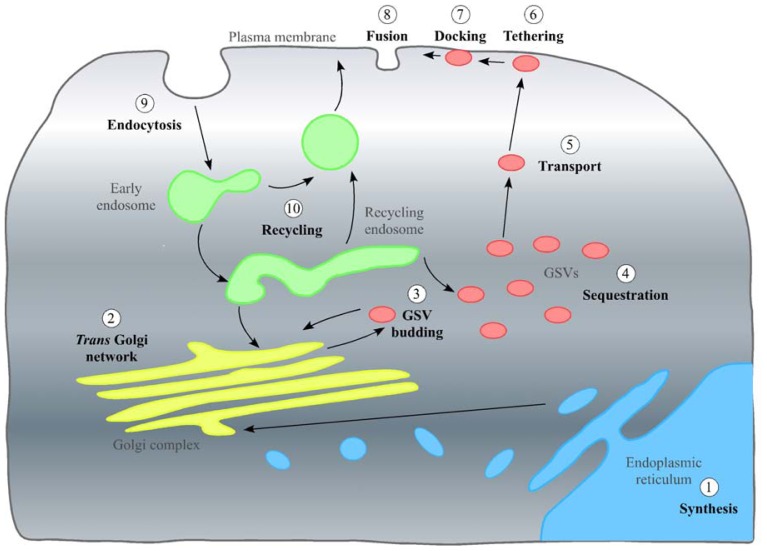
Schematic of the journey GLUT4 takes through different subcellular compartments. First, GLUT4 is translated within the endoplasmic reticulum (➀). GLUT4 is then transported to the *trans* Golgi network (➁), where it undergoes many post-translational modifications. GLUT4 is then inserted into budding GLUT4-storage vesicles (GSV) vesicles (➂). These vesicles are sequestered (➃) within the cell under basal conditions. This could be mediated by Tether containing UBX domain (TUG), which interacts with both GLUT4 in GSVs and the Golgi matrix protein, Golgin-160. Insulin stimulation releases GSVs from storage, and they are transported (➄) to the plasma membrane. Once GSVs arrive at the plasma membrane, they are tethered (➅), docked (➆) and, subsequently, fuse (➇) with the plasma membrane. GLUT4 inserted into the plasma membrane transports glucose into the cells down the concentration gradient. GLUT4 is also endocytosed (➈) and transported to the endosomal system for recycling (➉). This is either to the plasma membrane (if insulin is still present) or back to GSVs (if insulin is absent).

Figure 2 outlines the trafficking itinerary of GLUT4. Under basal (non-insulin stimulated) conditions, the majority of GLUT4 is retained intracellularly within two trafficking loops. The general endosomal recycling system serves to efficiently internalize GLUT4 cycling through early endosomes. From this cycle, GLUT4 enters a more specialized trafficking pathway that renders it insulin-sensitive. Critical to this pathway are small vesicles, 50–70 nm in diameter, termed GLUT4 storage vesicles (GSVs). It is from GSVs that GLUT4 is delivered to the plasma membrane (PM) in response to insulin. Biogenesis of GSVs and the sorting of GLUT4 into this insulin-sensitive store remains an area of intense research activity. Newly synthesized GLUT4 is thought to be transported through the *trans* Golgi network (TGN) and sequestered within GSVs without first cycling to the PM [6]. Delivery of GLUT4 to the PM in response to insulin increases glucose transport by between 10- and 30-fold. Once within the cell, glucose is either used as fuel or stored as triglycerides or glycogen, depending on cell type [7]. From the plasma membrane endocytosis internalizes GLUT4 to the early endosome. If insulin levels remain elevated, GLUT4 is recycled directly back to the PM. Once the insulin stimulation is removed, GLUT4 traffics to the recycling endosome. From here, it can either be targeted back to GSVs for storage or targeted for degradation in the lysosome. Some GLUT4 remains in the endosomal recycling system (estimated at around 40%) [8]. Given its physiological importance, the trafficking itinerary of GLUT4 is subject to multiple layers of regulation. Reviews of the machinery that controls GLUT4 trafficking can be found elsewhere [9–11]. This review will focus on the potential role that post-translational modifications of GLUT4 play in its sorting, subcellular localization and function.

Over 200 forms of post-translational modification have been identified globally, with this number growing annually, as computational analysis of proteins improves [12]. Defective insulin-stimulated translocation of GLUT4, as well as activity underlies the disease states of insulin-resistance and type 2 diabetes [13]. Disruption of post-translational modifications plays a role in many disease states [14–16], thus consideration of post-translational modification of GLUT4 may be important. We will now follow the journey of a GLUT4 molecule as it travels through multiple subcellular compartments (Figure 2), discussing evidence for post-translational modifications and the effects these have on localization and function.

## 2. In the Beginning: GLUT4 Synthesis, the TGN and Inclusion into GSVs

### 2.1. Stage 1: Synthesis and the Endoplasmic Reticulum

Proteins synthesis begins with transcription of DNA to mRNA; mRNA is then translated into a protein by ribosomes bound to the endoplasmic reticulum (ER). Almost 70% of proteins containing glycosylation consensus sites are glycosylated in the lumen of the ER [17]. Glycosylation is the addition of carbohydrate chains to proteins (or lipids) and is often required for correct protein folding and/or to improve protein stability [18]. Most GLUTs contain at least one glycosylation site within the exofacial loop (Figure 1) that is highly conserved (Asn-Xaa-Thr/Ser where X ≥ Pro) [17,19]. This site likely serves as the point of core glycosylation within the ER, which is then further modified as GLUT4 traffics through the Golgi apparatus. This was investigated by Williams *et al.*, who demonstrated that knockdown of golgin-160, a Golgi resident protein, is required for the proper trafficking of GLUT4 to GSVs. GLUT4 in these cells has an altered glycosylation profile. The intriguing observation here is that GLUT4 has lost its ability to be intracellularly retained, but rather accumulates on the cell surface. This suggests that either further glycosylation of GLUT4, after exit from the ER, is required for its proper intracellular sequestration or that Golgin-160 depletion disrupts GSV sequestration at a pre-Golgi compartment, preventing full glycosylation of GLUT4 [20].

### 2.2. Stage 2: The Trans Golgi Network

After transit through the Golgi stack, GLUT4 transits the TGN, where there is the potential for many post-translational modifications to occur, including sialylation, ubiquitination and SUMOylation. Once GLUT4 arrives at the TGN, the *N*-glycan chain may be further modified in a multistep process. This produces variations in both chain length and side chain branching, which affect the localization, function or stability of many proteins [17]. Within the TGN, GLUT4 is further glycosylated on Asn-57. This is important for protein stability. Studies characterizing the glycosylation defective GLUT4 Asn57Gln showed that levels of this mutant protein were 10-fold lower than wild-type GLUT4 in adipose cells [21]. This and other studies are consistent with the notion that the absence of the glycan drastically reduces GLUT4 protein stability [19,22].

Whilst in the TGN, GLUT4 undergoes other forms of post translational modification, including ubiquitination, SUMOylation, sialylation and possibly palmitoylation. The functional consequences of these post-translational modifications will be discussed below, as they likely impact mostly on the sorting of GLUT4 into GSVs.

### 2.3. Stage 3: Inclusion in GSVs

#### 2.3.1. Effects of Post-Translational Modifications on GLUT4 Interactions with Proteins Required for Sorting into GSVs

The insulin-responsive aminopeptidase (IRAP) and sortilin play an important role in the biogenesis of GSVs. Palmitoylation is required for retrograde trafficking of sortilin from the endosomal system through the TGN [23]. Ren and colleagues used a thiopropyl captivation approach to identify palmitoylated proteins in adipocytes. In this approach, all free cysteine residues are bound to methanethiosulfonate, and then the palmitic acid chains are removed using hydroxylamine. Following this, any free cysteine residues are captured by thiopropyl Sepharose, and any proteins unbound (*i.e.*, proteins not palmitoylated) are washed off; the identity of palmitoylated proteins can be determined through immunoblotting. They identified sortilin, GLUT4 and IRAP as being palmitoylated [24]. GLUT4 palmitoylation levels were increased in adipose tissue from obese and diabetic mice [24]. On the basis of these findings, it is tempting to speculate that palmitoylation plays a role in the sorting of proteins into GSVs. Since both sortilin and IRAP are required to be present at the site of GSV vesicle formation and sortilin palmitoylation is necessary for the trafficking of sortilin between the ER and the Golgi [23], palmitoylation may be required for the formation of the insulin responsive compartment. This has yet to be examined.

The number of genes encoding proteins involved in the addition of ubiquitin or ubiquitin-like modifiers (ULMs) to proteins is roughly equivalent to the number of genes involved in phosphorylation and dephosphorylation of proteins [25]. Ubiquitination is the addition of ubiquitin, a 76 amino acid protein, to the N termini of proteins or to Cys, Ser, Thr or Lys residues within a protein. This occurs in a multistep process, which sees an amide link forming between ubiquitin’s carboxy terminus and a primary amino group, often supplied by lysine residues [26]. Ubiquitin is highly conserved in eukaryotes with 96% sequence identity between human and yeast. One well characterized role of ubiquitin is to target proteins for degradation [27]. It has also been shown to play a role in internalization and trafficking of membrane proteins from the PM [28]. Ubiquitination of GLUT4 is required for sorting into GSVs [29]. Intriguingly, the Golgi-localized, γ-ear-containing, ADP ribosylation factor (ARF)-binding (GGA) family of ubiquitin-binding clathrin adaptor proteins have also been implicated in this sorting step, making it tempting to speculate that there is a link between these two observations [6,30].

SUMO-1, also known as Sentrin, is an ubiquitin like modifier (ULM). Ubiquitin conjugating enzyme 9 (Ubc9) is responsible for the covalent addition of SUMO-1 to proteins. SUMO-1 is 101 amino acid protein. SUMOylation has been found to regulate both protein subcellular localization and function [31–33]. As previously noted, SUMO-1 shares little sequence homology to ubiquitin (18%); however, its 3D structure is remarkably similar [25,34]. The role of SUMOylation in the acquisition of insulin responsiveness was studied by Liu *et al.* in 3T3-L1 cells. The GSV fraction can be separated from the endosomal/TGN fractions of adipocytes using iodoxinal gradient centrifugation. Ubc9 was found predominantly in the endosomal/TGN peak. This may suggest that SUMOylation may be required for targeting of GLUT4 to GSVs from either the TGN or the endosomal system [35]. The interplay between SUMOylation and ubiquitination remain to be defined.

#### 2.3.2. GLUT4 Insulin Responsiveness: When Are Modifications Needed?

The reason for sequestering GLUT4 into GSVs is to create a pool of GLUT4 that is responsive to insulin. Removing glycosylation has been shown to prevent the acquisition of insulin responsiveness [21]. This may be due to either a failure to produce GSVs or to an impaired ability to sense the insulin signal. When Ing *et al.* compared the insulin responsiveness of both wild-type GLUT4 and the GLUT4 Asn57Gln mutant, which remains in the un-glycosylated state, they found that cell surface levels of both proteins in the absence of insulin were similar in rat adipose cells. This suggests that under basal conditions both versions of GLUT4 are trafficked similarly. However, on stimulation with insulin, a 2.5-fold increase in wild-type GLUT4 levels at the cell surface was seen, while no significant increase was seen for GLUT4 Asn57Gln mutant [21]. This provided the first suggestion that N-glycosylation of GLUT4 may have a role in GLUT4 trafficking and sorting into GSVs. To further characterize the role of GLUT4 glycosylation, Haga *et al.*, 2011, looked at transport of GLUT4 in HeLa cells expressing either wild-type GLUT4 or GLUT4 Asn57Gln. HeLa cells have no endogenous GLUT4; they are, however, insulin responsive and are used as a model of ectopically expressed GLUT4 trafficking. In the absence of insulin, GLUT4 Asn57Gln was found at the cell surface at levels similar to its wild-type counterpart. In the presence of insulin, however, whereas levels of the wild-type protein at the cell surface increase dramatically, those of GLUT4 Asn57Gln do not. Furthermore, the wild-type and mutant GLUT4 have different subcellular localizations with more wild-type GLUT4 being found to co-localize with IRAP (indicating a GSV-like compartment) compared to the mutant [19]. This may indicate that *N*-glycosylation is required to facilitate interactions between GLUT4 and other GSV cargo proteins, such as sortilin in the vesicle lumen, supporting the theory that GSV cargos enter newly forming vesicles by mass action [36,37].

Some debate is still ongoing as to the role of glycosylation in the insulin responsiveness of GLUT4. Zaarour *et al.* report no significant difference between the trafficking of wild-type GLUT4 and GLUT4 Asn57Gln in pre-adipocytes, adipocytes, M6 myoblasts or HeLa cells. In their study, the mutant was found at the cell surface at similar levels to wild-type GLUT4 under both basal and insulin-stimulated conditions [22].

#### 2.3.3. Role of Post-translational Modifications on GLUT4 Protein Stability

As discussed above, post-translational modifications, such as *N*-glycosylation, can affect protein stability; any changes in GLUT4 protein stability are likely to affect GLUT4 protein content and, thus, insulin sensitivity. Interestingly, Zaarour *et al.* showed that the multiple band pattern generally seen for GLUT4 on immunoblots may not only represent different glycosylation states, but may also indicate multiple degradation products. Immunoblot analysis showed multiple bands for GLUT4 Asn57Gln, which cannot undergo glycosylation. This suggests that the multiple bands were not simply the product of multiple glycosylation forms [22].

SUMOylation may also improve GLUT4 stability, as in cells over-expressing Ubc9, a global increase in GLUT4 levels is evident. Pulse-chase experiments determined that GLUT4 is more stable in cells overexpressing Ubc9 than in control cells. As such, a small proportion of GLUT4 is SUMOylated at any one time; this may indicate that Ubc9 scaffolding and catalytic activities have a role in improving GLUT4 stability. To determine if this was due to improved sequestering of GLUT4 in GSVs, the half-life of GLUT4 was analysed in pre-adipocytes, which do not contain GSVs, and in mature adipocytes, which do. This indicated that increased stability arises from GLUT4 sorting into GSVs as GLUT4 was found to have a markedly longer half-life (50 h) in adipocytes compared to pre-adipocytes (5 h) [35].

#### 2.3.4. Effect on GSV Formation When Post-Translational Modifications Are Prevented

For SUMOylation to occur, Ubc9 must interact directly with the protein being modified. To determine if this interaction has a role other than the addition of SUMO-1, the interaction between GLUT4 and Ubc9 was studied in two ways. When wild-type Ubc9 was overexpressed in L6 skeletal muscle cells, there was an apparent eight-fold increase in GLUT4 cellular levels. However, when a catalytically inactive version of Ubc9 (Cys93Ala) was expressed, no such increase in GLUT4 abundance was observed. This suggests that the SUMOylation of GLUT4 was responsible for the elevated protein levels [38]. Secondly, depletion of Ubc9 by siRNA decrease GLUT4 levels ~50%. Less GLUT4 was found to be present in GSVs, and insulin-stimulated glucose transport was reduced. The knockdown also decreased the cellular levels of other TGN resident proteins, including syntaxin 6 and sortilin [35]. This suggests that Ubc9 may also play a secondary role in modulating GLUT4 levels, as decreasing the levels of syntaxin 6 and sortilin may decrease the number of GSVs present within a cell [30,35,39].

In addition to affecting its total cellular levels, SUMOylation also increases GLUT4 accumulation in GSVs, preventing it being trafficked to the PM via the endosomal system under basal conditions. When insulin was added, the fold increase in glucose transport was also markedly higher in cells overexpressing Ubc9 (3.3-fold compared to 1.8-fold), consistent with the notion that more GLUT4 is sequestered in GSVs. This effect requires the catalytic activity of Ubc9. This suggests that either SUMOylation or the enzymatic activity of Ubc9 is required for inclusion of GLUT4 into GSVs. Glucose transport after insulin stimulation was also 43% lower than seen in control cells, indicating a decreased pool of GLUT4 in GSVs [38].

#### 2.3.5. Effect of Increasing Levels of GLUT4 Post-translational Modification

Some forms of post-translational modification can undergo further modification to increase their complexity. For example, proteins with few glycosylation sites, such as GLUT4, are found at the cell surface more readily when chemicals increasing glycan branching are added [17].

Side chain branching can be achieved by adding acetylglucosamine (GlcNAc) to the media. In HEK293T cells, expressing GLUT4, grown in media containing insulin and GlcNAc higher levels of wild-type GLUT4 were found at the cell surface. These levels were found to increase in a manner dependent on the concentration of GlcNAc. When this effect was analysed in cells expressing GLUT4 Asn57Gln, which does not undergo *N*-glycosylation, the addition of GlcNAc had no effect on cell surface levels. This suggested that increasing side chain branching of the glycan was responsible for the improved mobility of GLUT4 in the wild-type expressing cells [17]. Glucosamine treatments have, however, previously been shown to induce cellular insulin resistance and block insulin responsive glucose transport [40,41]. Treatment with GlcNAc may therefore alter other cellular pathways, leading to increased levels of GLUT4 at the PM.

## 3. The Pause and Release: Sequestering of GSVs and Their Release on Stimulation with Insulin

### 3.1. Stage 4: Sequestering of GSVs

For GLUT4 to translocate to the PM in response to insulin, it must be segregated away from the endosomal recycling system. There is debate in the literature regarding whether this is a static or a dynamic retention mechanism [42–45]. Static retention states that GLUT4 is tethered to an intracellular anchor, and dynamic retention states that GLUT4 continually cycles between the GSV compartment and the endosomal recycling pool. One modification that has been proposed to be responsible for the static retention of GLUT4 has been tentatively called “TUGULation”. A proposed endoproteolytic cleavage site (diglycine motif found at residues 163 and 164) splits Tether containing UBX domain (TUG) into two fragments. The *N* terminal fragment (TUGUL) contains residues 1–164 and has some similarity to ubiquitin. TUG is thought to bind to GLUT4 while in GSVs and to also bind to a second protein that acts as an intracellular anchor (see below). The *N* and *C* terminal fragments are created when TUG is cleaved, which is proposed to occur upon insulin stimulation.

The interaction of the TUG ubiquitin-like domain (TUGUL) with GLUT4 was described by Bogan *et al.* [46]. In previous experiments, the authors had shown a direct interaction between the TUG protein and GLUT4. It was suggested that TUG may act as a putative regulator of GLUT4 traffic by forming a tether, preventing GSV release in the absence of insulin [47,48]. An interaction between TUG and the large intracellular loop of GLUT4, found between transmembrane domains VI and VII, was shown using a GST pull down assay [48]. Rough calculations suggest that there is approximately one molecule of TUG for each molecule of GLUT4 found in GSVs.

By using *N* and *C* terminal specific antibodies for TUG, products of multiple sizes were identified. Native TUG, detected by both the *N* and *C* terminal antibodies, was identified as a band of 60 kDa. The *N* terminal antibody only detected one other band of 130 kDa. The *C* terminal antibody detects proteins of 54 and 42 kDa, but was unable to detect the 130 kDa identified with the *N* terminal antibody. Creation of the 130 kDa and 54 kDa bands was thought to occur as a result of a single biochemical cleavage event. As yet, the protein(s) modified by the *N* terminal fragment of TUG remain unidentified, as pull down experiments using the TUG *N* terminal antibody failed [46]. One covalently-modified target of TUGULation was hypothesized to be a 110 kDa protein involved in the movement of GLUT4 from the perinuclear region to the cell periphery. One candidate protein is Kif5B, which acts in a wortmannin-insensitive manner (consistent with TC10a activation of TUG processing) [49]. Thus, TUG processing may transfer GLUT4 from Golgi matrix to microtubule motor proteins.

### 3.2. Stage 5: Transport of GLUT4 to the Cell Surface on Stimulation with Insulin

The *C* terminal region of TUG is thought to interact with both protein interacting specifically with Tc10 (PIST) and Golgin-160. PIST is an effector of the TC10 GTPase, which has been shown to play a role in GLUT4 translocation on stimulation with insulin [50]. Depletion of TC10α by RNAi prevents TUG cleavage and GSV release. Golgin-160 has already been implicated in GLUT4 trafficking [20]. PIST and a section of the TUG *C* terminus (377–550) have been shown to interact directly with Golgin-160. This complex may act as the cellular anchor for GSVs. On stimulation with insulin, TC10α may then cleave TUG, releasing GSVs, allowing them to translocate to the PM and increasing cell surface levels of GLUT4 [46].

The rate of TUG cleavage, analysed by pulse chase experiments, seemed to be accelerated in the presence of insulin. The cleavage products were also deemed to be adipocyte specific, as they were undetectable in 3T3-L1 pre-adipocytes and in HEK293 cells [46].

To assess if TUG cleavage was required for insulin-stimulated GLUT4 translocation, differential centrifugation was performed on cells containing wild-type TUG, TUG depleted by shRNA and a cleavage resistant form of TUG. In the first experiment using wild-type TUG, GLUT4 was found to translocate to the PM. When TUG was depleted by shRNA, this was abrogated. Cleavage-resistant TUG reduced GLUT4 translocation, suggesting that TUG cleavage, as well as TUGULation of GLUT4 is essential for GLUT4 translocation under insulin-stimulated conditions [46].

Another post-translational modification that has been proposed to regulate the translocation of GLUT4 in response to insulin is phosphorylation. Numerous putative phosphorylation sites exist on GLUT4 [51]. Two of these sites are Ser-274 and Ser-488 [52,53]. Under basal conditions, approximately 20% of the total pool of GLUT4 is phosphorylated [54] on Ser-488 [53]. Phosphorylation on Ser-274 has been shown to be required for glucose uptake when GLUT4 and the respective kinase (SGK1) are expressed in oocytes [52]. In adipocytes, SGK1 is stimulated by PI3K in response to insulin, indicating that the phosphorylation of Ser-274 may be involved in the insulin response. However, as yet, no functional role for phosphorylation on Ser-274 has been shown in adipocytes.

It has been proposed that insulin stimulation results in dephosphorylation of GLUT4 and this triggers its movement to the cell surface. This comes from evidence showing that okadaic acid, an inhibitor of phosphatases, attenuates the insulin-stimulated increase in glucose uptake [55]. Dephosphorylation of GLUT4 in response to insulin has indeed been shown in a number of studies. At first glance, the immunoblots in these papers appear to show an increase in phosphorylation of GLUT4 in the cell surface fraction, but when this is normalized to the total amount of GLUT4 in the fraction, there is actually a reduction in phosphorylation [53,56].

A number of studies, however, have shown that there is no change in phosphorylation [53,54], and one study found that insulin actually increased GLUT4 phosphorylation [57]. In 1999, Kupriyanova *et al.* found that insulin stimulation for 15 min increased GLUT4 phosphorylation [57]. After examining this more closely, they found that at two minutes of insulin stimulation, there was a sharp increase in GLUT4 phosphorylation and that GLUT4 phosphorylation levels fell again up to 15 min, at which point measurement was stopped. The previously mentioned studies that reported decreased phosphorylation of GLUT4 administered insulin between 20 and 30 min. It is therefore possible that insulin may cause an initial increase in phosphorylation, followed by a reduction of phosphorylation.

If phosphorylation was involved in the translocation of GLUT4 from intracellular deposits, increasing phosphorylation should increase glucose uptake; this, however, is not the case. Addition of Isoproterenol, which stimulates phosphorylation of GLUT4, decreased glucose uptake levels in adipocytes, even though GLUT4 appeared to translocate to the cell surface as normal [53,54]. However, subsequent studies have proposed that isoproterenol administration leads to the accumulation of docked, but not fused GLUT4-containing vesicles beneath the PM [58,59]. The role of phosphorylation in this process remains unstudied. Similarly, if dephosphorylation was a trigger for GLUT4 translocation to the cell surface in response to insulin, phosphorylated GLUT4 would not be expected to translocate. This is not the case, as insulin increases the absolute amount of phosphorylated GLUT4 in the cell surface fraction (*i.e.*, insulin induced translocation of phosphorylated GLUT4) [53]. Consequently, phosphorylation may not be involved in the translocation of GLUT4 to the cell surface in response to insulin. These findings have led to the proposition that dephosphorylation of GLUT4 at the cell surface may trigger endocytosis [55], which is discussed in more detail in Section 5.1.

There is some evidence that GLUT4 phosphorylation status is altered in diabetes. Two *in vitro* models of diabetes in primary adipocytes show increased phosphorylation of GLUT4 [56,60]. The elevated phosphorylation of GLUT4 seems to be a result of changes in GLUT4 structure, rather than a change in the phosphatases that regulate the balance of phosphorylated to dephosphorylated GLUT4 [60]. The anti-diabetic drug, Glimepiride, increases glucose uptake in insulin resistant adipocytes by inducing GLUT4 translocation, whilst concurrently decreasing GLUT4 phosphorylation [56]. It is not clear if this reduction in GLUT4 phosphorylation leads to an increase in insulin sensitivity. The data suggest that increased GLUT4 phosphorylation impairs insulin responsiveness in diabetic adipose tissue. In support of this model, elevating GLUT4 phosphorylation by increasing intracellular calcium levels impairs the insulin response. Furthermore, increases in insulin induced glucose uptake can be recovered by using cAMP antagonists, which reduce GLUT4 phosphorylation [61].

## 4. Fulfilling Its Purpose: Stages 6–8, GLUT4 Insertion into the PM and Its Function as a Glucose Transporter

Once GLUT4 has been transported and inserted into the PM, its sole function is to transport glucose into the cells down the concentration gradient. Under basal conditions, a small amount of GLUT4 is found at the PM, allowing low levels of glucose uptake. Post-translational modifications affecting GLUT4 inclusion into GSVs can affect the levels of glucose transport in both the basal and insulin stimulated states, while having no effect on the ability of GLUT4 to transport insulin.

For example, although GLUT4 glycosylation affects the insulin responsiveness of GLUT4, it does not affect its ability to function as a glucose transporter. The glycosylation deficient GLUT4 mutant, Asn57Gln, is not packaged into GSVs and does not translocate to the PM on stimulation as efficiently as wild-type GLUT4; yet, when it does translocate, it still functions as a glucose transporter. This was shown using uptake of a fluorescence glucose analogue as a marker. The level of uptake was above background rates in both wild-type and GLUT4 Asn57Gln expressing HeLa cells when stimulated with insulin [19,22].

## 5. Re-Entry: Endocytosis and Transport of GLUT4 through the Endosome System

### 5.1. Stage 9: Endocytosis of GLUT4

Endocytosis is a continuous process internalizing PM proteins, including GLUT4. This occurs both in the presence and absence of insulin, but the rate of endocytosis may be affected by the post-translational modification state of GLUT4.

The fluorescence resonance energy transfer (FRET) technique has been applied to study internalization of GLUT4 in HeLa cells [62]. In this study, glycans were metabolically labelled with azide sugars (in their case, azide-tagged sialic acid), and they were further conjugated with a fluorophore (such as Alexa Fluor 555) by click chemistry. The target protein is conjugated with GFP. Then, the specific glycoprotein can be visualized by FRET between GFP and the glycan-conjugated fluorophore. While the GFP signal represents total GLUT4 (both internal and extracellular), the FRET signal represents the sialylated GLUT4, most of which is localized on the cell surface (as fluorophore-labelling was carried out under conditions to minimize the labelling of intracellular azide-tagged sialic acid). It is therefore possible to monitor, upon the removal of insulin from the media, internalization of FRET signal (sialylated GLUT4) and compare it to that of overall GLUT4 (GFP signal) using a special microscopy, called TIRF (total internal reflection fluorescence) [62]. While using GLUT4 as a model PM protein, Haga *et al.* [62] showed that modification of the glycan chain with azide-tagged sialic acid caused GLUT4 to be internalized at a slower rate in the absence of insulin compared to untreated GLUT4. When performing the same experiment with the GLUT4 Asn57Gln mutant, which lacks a glycan chain suitable for azide-tagged sialic acid addition, the internalization rate was unchanged in modified and unmodified conditions. This suggests that modifications of the Glycan chain added during *N*-glycosylation affect the rate of GLUT4 internalization [62].

The effect of *N*-glycosylation on GLUT4 endocytosis has also been analysed using a more standard protocol. Zaarour *et al.* used an antibody internalization experiment to determine if *N*-glycosylation affected the rate of GLUT4 internalization. In this experiment, HA-tagged GLUT4 or GLUT4 Asn57Gln was used. These experiments found no difference in cell surface recycling for either basal or insulin induced conditions for either wild-type GLUT4 or GLUT4 Asn57Gln [22].

It has been proposed that the phosphorylation status of GLUT4 plays a role in endocytosis. In support of this, treatment with okadaic acid, which prevents GLUT4 dephosphorylation, slowly increases GLUT4 at the cell surface, which could be due to the inhibition of GLUT4 internalization [55].

In an attempt to examine the role of GLUT4 phosphorylation in the endocytosis and sorting of GLUT4 into the insulin sensitive compartment, Marsh *et al.*, 1998, mutated Ser-488 to Ala (SAG mutant) [63]. They found that GLUT4 phosphorylation was abolished, and although there was no impairment in the insulin response, recycling of GLUT4 into the endosomal compartment was slightly slower. These data support the model by which dephosphorylation plays a role in triggering endocytosis.

### 5.2. Stage 10: The End

Once GLUT4 has been internalized, it travels through the endosomal system. From the endosomal system, GLUT4 has two options. It can either be shuttled back to the PM or be shuttled back to GSVs for storage. When the insulin signal is still present, the majority of GLUT4 is shuttled back to the PM. This allows glucose uptake to be maintained at a high rate until blood glucose levels drop. In the absence of an insulin signal, the majority of GLUT4 is transported back to the TGN, where it is re-packaged into GSVs for storage. A single GLUT4 molecule is likely to undergo multiple recycling events during the course of its lifetime. Once a GLUT4 molecule has reached the end of its lifespan, a third option becomes available. This is degradation by the ubiquitin-proteasome system or by lysosomes [35]. In these systems, GLUT4 is degraded and its components recycled to create new proteins, restarting the cycle from the beginning.

## 6. Summary

Post-translational modifications of GLUT4 are important for cellular localization. Alteration of this localization can indirectly affect insulin responsiveness of GLUT4, impairing glucose transport and possibly contributing to the development of insulin insensitivity and type 2 diabetes. Post translational modifications of proteins occur in specific compartments within the cell. Some proteins require post-translational modifications at some residues before a second modification can be made. To further our understanding of GLUT4 post-translational modifications and their roles in GLUT4 translocation and sorting the order in which these modifications occur needs to be studied. This would allow the creation of a more detailed map of each modification.

## References

[1] Bogan J.S. (2012). Regulation of glucose transporter translocation in health and diabetes. Annu. Rev. Biochem.

[2] Birnbaum M.J. (1989). Identification of a novel gene encoding an insulin responsive glucose transporter protein. Cell.

[3] James D.E., Strube M., Mueckler M. (1989). Molecular cloning and characterization of an insulin-regulatable glucose transporter. Nature.

[4] Pessin J.E., Bell G.I. (1992). Mammalian facilitative glucose transporter family structure and molecular regulation. Annu. Rev. Physiol.

[5] Huang S., Czech M.P. (2007). The glut4 glucose transporter. Cell Metab.

[6] Watson R.T., Khan A.H., Furukawa M., Hou J.C., Li L., Kanzaki M., Okada S., Kandror K.V., Pessin J.E. (2004). Entry of newly synthesized glut4 into the insulin-responsive storage compartment is gga dependent. EMBO J.

[7] Saltiel A.R., Kahn C.R. (2001). Insulin signalling and the regulation of glucose and lipid metabolism. Nature.

[8] Livingstone C., James D.E., Rice J.E., Hanpeter D., Gould G. (1996). Compartment ablation analysis of the insulin-responsive glucose transporter (glut4) in 3t3-l1 adipocytes. Biochem. J.

[9] Bryant N.J., Gould G.W. (2011). Snare proteins underpin insulin-regulated glut4 traffic. Traffic.

[10] Stöckli J., Fazakerley D.J., James D.E. (2011). Glut4 exocytosis. J. Cell Sci.

[11] Leto D., Saltiel A.R. (2012). Regulation of glucose transport by insulin traffic control of glut4. Nat. Rev. Mol. Cell Biol.

[12] Li S., Iakoucheva L.M., Mooney S.D., Radivojac P. (2010). Loss of Post-translational Modification Sites in Disease. Proceedings of the Pacific Symposium on Biocomputing.

[13] Garvey W.T., Huecksteadt T.P., Matthaei S., Olefsky J.M. (1988). Role of glucose transporters in the cellular insulin resistance of type ii non-insulin-dependent diabetes mellitus. J. Clin. Investig.

[14] Hardy J. (1997). Amyloid, the presenilins and Alzheimer’s disease. Trends Neurol. Sci.

[15] Toh K.L., Jones C.R., He Y., Eide E.J., Hinz W.A., Virshup D.M., Ptácek L.J., Fu Y.H. (2001). An hper2 phosphorylation site mutation in familial advanced sleep phase syndrome. Science.

[16] Grasbon-Frodl E., Lorenz H., Mann U., Nitsch R.M., Windl O., Kretzschmar H.A. (2004). Loss of glycosylation associated with the t183a mutation in human prion disease. Acta Neuropathol.

[17] Lau K.S., Partridge E.A., Grigorian A., Silvescu C.I., Reinhold V.N., Demetriou M., Dennis J.W. (2007). Complex *n*-glycan number and degree of branching cooperate to regulate cell proliferation and differentiation. Cell.

[18] Varki A., Cummings R.D., Esko J.D., Freeze H.H., Stanley P., Bertozzi C.R., Hart G.W., Etzler M.E. (2009). Essentials of Glycobiology.

[19] Haga Y., Ishii K., Suzuki T. (2011). *N*-glycosylation is critical for the stability and intracellular trafficking of glucose transporter glut4. J. Biol. Chem.

[20] Williams D., Hicks S.W., Machamer C.E., Pessin J.E. (2006). Golgin-160 is required for the golgi membrane sorting of the insulin-responsive glucose transporter glut4 in adipocytes. Mol. Biol. Cell.

[21] Ing B.L., Chen H., Robinson K.A., Buse M.G., Quon M.J. (1996). Characterization of a mutant glut4 lacking the *N*-glycosylation site studies in transfected rat adipose cells. Biochem. Biophys. Res. Commun.

[22] Zaarour N., Berenguer M., Le Marchand-Brustel Y., Govers R. (2012). Deciphering the role of glut4 *N*-glycosylation in adipocyte and muscle cell models. Biochem. J.

[23] McCormick P.J., Dumaresq-Doiron K., Pluviose A.S., Pichette V., Tosato G., Lefrancois S. (2008). Palmitoylation controls recycling in lysosomal sorting and trafficking. Traffic.

[24] Ren W., Jhala U., Du K. (2013). Proteomic analysis of protein palmitoylation in adipocytes. Adipocyte.

[25] Herrmann J., Lerman L.O., Lerman A. (2007). Ubiquitin and ubiquitin-like proteins in protein regulation. Circ. Res.

[26] Urbe S. (2005). Ubiquitin and endocytic protein sorting. Essays Biochem.

[27] Piper R.C., Luzio J.P. (2007). Ubiquitin-dependent sorting of integralmembrane proteins for degradation in lysosomes. Curr. Opin. Cell Biol.

[28] Hicke L. (2001). Protein regulation by monoubiquitin. Nat. Rev. Mol. Cell Biol.

[29] Lamb C.A., McCann R.K., Stockli J., James D.E., Bryant N.J. (2010). Insulin-regulated trafficking of glut4 requires ubiquitination. Traffic.

[30] Shi J., Kandror K.V. (2005). Sortilin is essential and sufficient for the formation of glut4 storage vesicles in 3t3-l1 adipocytes. Dev. Cell.

[31] Matunis M.J., Coutavas E., Blobel G. (1996). A novel ubiquitin-like modification modulates the partitioning of the ran-gtpase-activating protein rangap1 between the cytosol and the nuclear pore complex. J. Cell Biol.

[32] Mahajan R., Delphin C., Guan T., Gerace L., Melchior F. (1997). A small ubiquitin-related polypeptide involved in targeting rangap1 to nuclear pore complex protein ranbp2. Cell.

[33] Gill G. (2005). Something about sumo inhibits transcription. Curr. Opin. Genet. Dev.

[34] Bayer P., Arndt A., Metzger S., Mahajan R., Melchior F., Jaenicke R., Becker J. (1998). Structure determination of the small ubiquitin-related modifier sumo-1. J. Mol. Biol.

[35] Liu L., Omata W., Kojima I., Shibata H. (2007). The sumo conjugating enzyme ubc9 is a regulator of glut4 turnover and targeting to the insulin-responsive storage compartment in 3t3-l1 adipocytes. Diabetes.

[36] Pilch P.F. (2008). The mass action hypothesis: Formation of glut4 storage vesicles, a tissue-specific, regulated exocytic compartment. Acta Physiol.

[37] Shi J., Kandror K.V. (2007). The luminal vps10p domain of sortilin plays the predominant role in targeting to insulin-responsive glut4-containing vesicles. J. Biol. Chem.

[38] Giorgino F., de Robertis O., Laviola L., Montrone C., Perrini S., McCowen K.C., Smith R.J. (2000). The sentrin-conjugating enzyme mubc9 interacts with glut4 and glut1 glucose transporters and regulates transporter levels in skeletal muscle cells. Proc. Natl. Acad. Sci. USA.

[39] Perera H.K.I., Clarke M., Morris N.J., Hong W., Chamberlain L.H., Gould G.W. (2003). Syntaxin 6 regulates glut4 trafficking in 3t3-l1 adipocytes. Mol. Biol. Cell.

[40] Marshall S., Bacote V., Traxingerg R.R. (1991). Discovery of a metabolic pathway mediating glucose-induced desensitization of the glucose transport system; role of hexosamine biosynthesis in the induction of insulin resistance. J. Biol. Chem.

[41] Marshall S., Nadeau O., Yamasaki K. (2005). Glucosamine-induced activation of glycogen biosynthesis in isolated adipocytes: Evidence for a rapid allosteric control mechanism within the hexosamine biosynthesis pathway. J. Biol. Chem.

[42] Fujita H., Hatakeyama H., Watanabe T.M., Sato M., Higuchi H., Kanzaki M. (2010). Identification of three distinct functional sites of insulin-mediated glut4 trafficking in adipocytes using quantitative single molecule imaging. Mol. Biol. Cell.

[43] Govers R., Coster A.C.F., James D.E. (2004). Insulin increases cell surface glut4 levels by dose dependent discharging glut4 into a cell surface recycling pathway. Mol. Cell. Biol.

[44] Martin O.J., Lee A., McGraw T.E. (2006). Glut4 distribution between the plasma membrane and the intracellular compartments is maintained by an insulinmodulated bipartite dynamic mechanism. J. Biol. Chem.

[45] Karylowski O., Zeigerer A., Cohen A., McGraw T.E. (2004). Glut4 is retained by an intracellular cycle of vesicle formation and fusion with endosomes. Mol. Biol. Cell.

[46] Bogan J.S., Rubin B.R., Yu C., Löffler M.G., Orme C.M., Belman J.P., McNally L.J., Hao M., Cresswell J.A. (2012). Endoproteolytic cleavage of tug regulates glut4 glucose transporter translocation. J. Biol. Chem.

[47] Bogan J.S., Hendon N., McKee A.E., Tsao T.S., Lodish H.F. (2003). Functional cloning of tug as a regulator of glut4 glucose transporter trafficking. Nature.

[48] Yu C., Cresswell J., Loffler M.G., Bogan J.S. (2007). The glucose transporter 4-regulating protein tug is essential for highly insulin-responsive glucose uptake in 3t3-l1 adipocytes. J. Biol. Chem.

[49] Semiz S., Park J.G., Nicoloro S.M.C., Furcinitti P., Zhang C., Chawla A., Leszyk J., Czech M.P. (2003). Conventional kinesin kif5b mediates insulinstimulated glut4 movements on microtubules. EMBO J.

[50] Chang L., Chiang S.H., Saltiel A.R. (2007). Tc10alpha is required for insulin-stimulated glucose uptake in adipocytes. Endocrinology.

[51] Phosphosite.

[52] Jeyaraj S., Boehmer C., Lang F., Palmada M. (2007). Role of sgk1 kinase in regulating glucose transport via glucose transporter glut4. Biochem. Biophys. Res. Commun.

[53] Lawrence J.C., Hiken J.F., James D.E. (1990). Phosphorylation of the glucose transporter in rat adipocytes. Identification of the intracellular domain at the carboxyl terminus as a target for phosphorylation in intact-cells *in vitro*. J. Biol. Chem.

[54] James D.E., Hiken J., Lawrence J.C. (1989). Isoproterenol stimulates phosphorylation of the insulin-regulatable glucose transporter in rat adipocytes. Proc. Natl. Acad. Sci. USA.

[55] Lawrence J.C., Hiken J.F., James D.E. (1990). Stimulation of glucose transport and glucose transporter phosphorylation by okadaic acid in rat adipocytes. J. Biol. Chem.

[56] Muller G., Wied S. (1993). The sulfonylurea drug, glimepiride, stimulates glucose transport, glucose transporter translocation, and dephosphorylation in insulin-resistant rat adipocytes *in vitro*. Diabetes.

[57] Kupriyanova T.A., Kandror K.V. (1999). Akt-2 binds to glut4-containing vesicles and phosphorylates their component proteins in response to insulin. J. Biol. Chem.

[58] Vannucci S.J., Nishimura H., Satoh S., Cushman S.W., Holman G.D., Simpson I.A. (1992). Cell surface accessibility of glut4 glucose transporters in insulin-stimulated rat adipose cells: Modulation by isoprenaline and adenosine. Biochem. J.

[59] Yang J., Hodel A., Holman G.D. (2002). Insulin and isoproterenol have opposing roles in the maintenance of cytosol ph and optimal fusion of glut4 vesicles with the plasma membrane. J. Biol. Chem.

[60] Begum N., Draznin B. (1992). Effect of streptozotocin-induced diabetes on glut-4 phosphorylation in rat adipocytes. J. Clin. Invest.

[61] Reusch J.E., Begum N., Sussman K.E., Draznin B. (1991). Regulation of glut-4 phosphorylation by intracellular calcium in adipocytes. Endocrinology.

[62] Haga Y., Ishii K., Hibino K., Sako Y., Ito Y., Taniguchi N., Suzuki T. (2012). Visualizing specific protein glycoforms by transmembrane fluorescence resonance energy transfer. Nat. Commun..

[63] Marsh B.J., Martin S., Melvin D.R., Martin L.B., Alm R.A., Gould G.W., James D.E. Mutational analysis of the carboxy-terminal phosphorylation site of glut-4 in 3t3-l1 adipocytes.

